# Bioactivity of selected materials for coffee substitute

**DOI:** 10.1371/journal.pone.0206762

**Published:** 2018-11-15

**Authors:** Renata Zawirska-Wojtasiak, Paulina Piechowska, Elżbieta Wojtowicz, Krzysztof Przygoński, Sylwia Mildner-Szkudlarz

**Affiliations:** 1 Faculty of Food Science and Nutrition, Poznań University of Life Sciences, Poznań, Poland; 2 Department of Food Concentrates and Starch Products, Institute of Agricultural and Food Biotechnology, Poznań, Poland; College of Agricultural Sciences, UNITED STATES

## Abstract

Epidemiological studies have suggested that coffee consumption is negatively correlated with the incidence of Parkinson’s disease. Coffee contains relatively high levels of β-carbolines, which have been ascribed neuroactive effects in humans however the positive or negative effect has not been confirmed yet. Two ingredients with applications as coffee substitutes—chicory, which is traditionally used in this way, and artichoke—were considered in this study both from the neuroactive point of view but also in relation to the other bioactive compounds that result from their thermal processing. These thermal products are of concern because of their possible toxic properties. The estimated concentration of β-carbolines was high in both materials (1.8 μg/g and 2.5 μg/g harman and 2.9 μg/g and 3.1 μg/g norharman in chicory and artichoke, respectively). Artichoke had more β-carbolines than chicory, and also more all the toxic compounds examined here–acrylamide, carboxymethyllysine, and furans, which were detected in significantly higher concentrations in artichoke, particularly acrylamide. Chicory and artichoke also contain phenolic compounds that possess high antioxidant activity, on a similar level. Artichoke, a new proposed ingredient in coffee substitutes, appears to be a richer source of β-carbolines than the traditionally chicory. Both materials contained high level of undesirable components, such as furan and its derivatives, carboxymethyllysine and particularly acrylamide, much higher in artichoke.

## Introduction

Coffee is the third most popular beverage, after water and tea. Annual coffee consumption is estimated at 400 billion cups, and it is drunk by 40% of the human population. Its popularity is associated with its unique aroma and stimulating properties. Epidemiological studies suggest that coffee consumption is negatively correlated with the incidence of Parkinson’s disease, and that its regular consumption may protect to a certain degree against neurodegenerative diseases. This protective effect is ascribed to the presence of β-carboline compounds [1, 2]. The first report on the identification, quantification and formation of β-carboline compounds was published by Herraiz [3], who pointed out coffee as the highest dietary source of these compounds. Other studies report that elevated levels of harman and norharman occur in the cerebrospinal fluid of patients suffering from Parkinson’s disease [4]. Also later papers mention the possible neurotoxity of β-carbolines [5, 6].

Due to its properties and caffeine content, not everyone can consume coffee. The food industry offers a number of coffee substitutes (sometimes called grain coffees). These are usually produced from roast chicory, grains, and beetroot, as well as other new components, such as artichokes [7, 8]. Coffee substitute is becoming increasingly popular as a wellness product.

Research has recently suggested the presence of undesirable or even toxic compounds in various foodstuffs [9, 10]. Many of these compounds are created during the food production process, and particularly during thermal processing, which means that coffee substitute may also be affected. Morehouse *et al*. [11] have suggested the thermal degradation and rearrangement of organic compounds, particularly of carbohydrates, as probable mechanisms for furan formation in food. Furans are common products of the Maillard reaction (MR), the source of many key compounds of processed food aroma [12]. Some of these compounds have been questioned because of their possible toxic properties or their other negative effects on the human body. These compounds include acrylamide (ACR) [13,14], carboxymethyllysine (CML) [15] and furan and its derivatives [16, 17]. Literature sources have suggested that furans may exhibit toxic properties, and they have received a great deal of attention, as they are classified as potential carcinogens in humans [18]. The roasting process involved in producing both true coffee and coffee substitutes can lead to the formation of large amounts of furans. The concentration of furans in coffee is higher than in other processed food products [19, 20]. Recent studies suggest that furans may be responsible for cancer [16, 21]. A comprehensive study performed by the FDA found furan levels of up to 100 ppb in various food samples [18]. According to Morehouse *et al*. [11], furan is a suspected human carcinogen that is formed in some processed food and is found at levels ranging from not detectable (limit of detection LOD, 0.2–0.9 ng/g) to over 100 ng/g, while the daily intake for consumers is estimated to be about 0.2 μg/kg body weight (bwt). Much higher doses, such as 2 mg furan/kg bwt, may induce tumor in mice, a result that can be extrapolated from animal experiments to humans with at least with some certainty [22]. Other than coffee [19, 23, 24] other processed foods in which furans commonly occur are breakfast cereals [24], cakes [19, 24], bread [25] and roast chicory [26]. Besides furans, alkylated furans have also been detected in heat-processed food, including coffee [19], as have other furan derivatives (furanic compounds), such as furfural [17, 27, 28] and especially 5-hydroxymethylfurfural (HMF). This last compound, which has been ascribed mutagenic properties, has been found in large amounts in coffee [29] as well as in coffee substitutes [30]. Thermal processes in food may lead to the formation of other undesired substances, one of which is CML [15, 31, 32]. This compound is derived from lysine and has been associated with pathogenic processes in some diseases of civilization. It has been found in chocolate powders, and also measured in coffee at a concentration of 0.17 to 47 mg/kg—the higher value related to instant coffee [30].

Another decidedly undesirable compound in food is ACR [14, 33]. Currently, the concentration of ACR in processed food products has become a very serious health issue [13], as in the laboratory it causes tumors in animals. The problem of high ACR concentrations also arises in coffee and coffee substitutes [30, 34]. The literature indicates that the concentration of ACR in coffee substitutes is much higher than in true coffee, by a factor of perhaps two or three. The value varies widely but has been found to reach as high as 818 μg/kg [34] or even 4940 μg/kg [30].

Given the occurrence of these various bioactive components, the question arises of the quantitative relations between these compounds in coffee and its substitutes. The number and concentration of harmful components may suggests to avoid of drinking them, thus limiting consumption of compounds, such as furan and its derivatives, CML, ACR and might be also β-carbolines.

It is worth noting that many MR products possess high antioxidant activity (AOA), which may be beneficial to human health [35] and which may also act against the formation of CML [15]. This is particularly true in a material such as chicory, which is regarded as a source of dietary antioxidants [36]. One such compounds, chlorogenic acid, has been found in large amounts in the raw ingredients of coffee substitutes, but was significantly lost during roasting [7]. The total phenols (TP), ABTS, and DPPH tests, as well as measurement of the concentration of antioxidant polyphenol, phenolic acids, and flavonoids, have also been considered in the context of thermal product characteristics.

From the toxicology point of view, it is not the fact that a compound is present that is of significance, but its concentration. The aim of this study was thus to estimate the concentration of furans, CML, and ACR and neuroactive β-carbolines in roast chicory and artichoke as an example of one possible additional raw materials, and in mixtures of both. Artichoke was selected as this of β-carbolines concentration potential. These two ingredients respectively represent the traditional raw material and a possible new ingredient for coffee substitutes.

## Materials and methods

### Sample preparation

Chicory (*Cichorium intybus*) and artichoke (*Cynara scolymus* L.) were used in this study. Dried chicory was obtained from production plant of Cykoria S.A. (Wierzchosławice, Poland). Artichokes were purchased from local supermarket. Their hearts were isolated and dried in a laboratory drier at 60°C. Roast chicory, roast artichoke, and mixtures of both at 10%, 20%, and 30% artichoke (Mix I, Mix II, and Mix III respectively), were prepared. Before and after roasting, the chicory and artichoke were ground using a WZ1 laboratory mill (Sadkiewicz Instruments, Bydgoszcz, Poland). The roasting process was carried out in a Probat BRZ2/4/6 sample roaster battery (Emmerich am Rhein, Germany). The samples of 100 g each were roasted at 160 °C for 8 min (artichoke) and 180 °C for 10 min (chicory) to obtain dark roasts.

Beverages were prepared from these materials following the SCAA Standard Golden Cup [37] (55 g/L ± 10%—meaning about 5 g coffee per 100 mL water).

### Beta-carbolines assay

The norharman and harman contents were assayed using a method described by Alves *et al*. [1], modified for the study materials according to our previous work [8]. High-performance liquid chromatography (HPLC) was used to analyze the β-carboline content using a Dionex Corporation (Sunnyvale, CA, USA) LC system equipped with an autosampler and an RF-200 fluorescence detector with a Supelco (Bellefonte, PA, USA) C18 column (150 × 4.6 mm, 100 Å, 3 μm). The wavelengths of fluorescence detector were respectively, 300 and 440 nm for excitation and emission. Standards of harman and norharman were provided from Fluka (Steinhem, Germany) and Sigma Aldrich (Steinham, Germany), respectively.

### Determination of furan and furan derivatives

Five grams of samples were weighed into a 20-mL vial and followed by 1 mL of distilled water and 100 μL of labeled standard ^2^H_4_ furan solution, providing a sample concentration of 200 ng/g. To optimize the extraction process with fiber (SPME Fiber Assembly 75 μm Carboxen–PDMS from Supelco Bellefonte, PA, USA), the exposure was performed at various temperatures (40 °C, 50 °C, 60 °C, and 70 °C) and for various durations (10, 20, 30, and 40 min). The best results—good resolution and the highest peak area—were achieved at 50 °C after 30 min, and those conditions were therefore employed. The fiber was then removed from the vial to the injection port of the gas chromatograph for 5 min of desorption.

GC/MS analysis was performed using an Agilent 7890A gas chromatograph connected to an MS detector Agilent 5957C VLMSD. Compounds were separated on a Supelco SLB-5ms nonpolar column using the following temperature program: from 45 °C (hold 2 min) to 150 °C at a rate of 5 °C/min, then to 190 °C at a rate of 9 °C/min, and finally to 230 °C at a rate of 18 °C/min (held 5 min). The carrier gas was helium, the detector temperature was 270 °C, and the injector temperature was 220 °C.

The separated compounds were identified by comparing their retention indices (RI) and mass spectra with standards (furan, 2-methylfuran, 2,5-dimethylfuran, > 99%) from Sigma Aldrich, Steinham, Germany or, in some cases, tentatively only by the NIST MS Search 2.0 mass spectra library search, considering those with spectrum similarity over 70%. Quantitative calculations were performed taking into account known amounts of the added internal standard ^2^H_4_ furan (98% atom D, Sigma Aldrich, Steinham, Germany), as well as the *m/z* 43 and *m/z* 72 ion response ratio [38]. Measurements were performed with five repetitions. Using the same procedure, the other furans and furfural were identified.

### Assay of CML

The procedure for measuring CML followed a method that has been described earlier [15]. Following defatting, protein reduction, hydrolysis, and derivatization using *o*-phthaldialdehyde, CML determination was performed using HPLC (Waters, Milord, MA, USA) and a Waters Sun Fire C18 column (150 × 4.6 mm, 5 μm; Milord, MA, USA). A gradient program was used, combining solvent A (acetate buffer and acetonitrile 9:1, v/v) and solvent B (50% acetonitrile) as follows: 99%–70% A (25 min), 70%–99% A (15 min). The flow rate was 1.0 mL/min, the injection volume was 10 μL, and the column temperature was maintained at 20 °C. Detection was at 340 nm (excitation) and 455 nm (emission). The peaks for CML derivatives in the samples were confirmed by comparison with an authentic sample of CML provided by PolyPeptide Laboratories France SAS (Strasbourg, France). The compounds identified were quantified using the external standard calibration procedure.

### Acrylamide quantification by LC-MS/MS

Acrylamide estimation was performed using gas-liquid chromatography method with tandem mass spectrometry (LC-MS/MS). 0.5 g of artichoke and 1.5 g of chicory and mix samples were weight for analysis. To each sample internal standard solution was added (2,3,3-d_3_ acrylamide 98% atom D from Sigma Aldrich, Steinham, Germany; AAd_3_ in concentration of 1000 μg/L): 0.3 ml to artichoke and 2.0 mL to all the rest samples. Acrylamide was extracted with water, than purified in 3 steps procedure: hexane, Carreze I and II solution and finally on SPE columns. More details is described by Mojska and Gielecińska [34]. Data were read out from matrix curve prepared for acrylamide solutions (5 do 1000 μg/L) with addition of internal standard.

### Measuring antioxidant activity

#### Preparation of samples

The ground materials (0.5 g) were extracted with 10 mL of water:acetone mixture (3:7, v:v) in a mechanical shaker for 60 min and then centrifuged (10 min, 3000 rpm). The supernatant was transferred to a 25 mL extraction flask and the extraction was repeated with a methanol:water mixture (7: 3, v:v). The supernatants were combined and filled to the mark with a acetone:water:methanol mixture (7:7:6, v:v:v).

#### ABTS

Free radical scavenging activity was determined by ABTS radical cation decolorization assay, as described by Re *et al*. [39]. Results were expressed as Trolox equivalents (TxE). The analysis were repeated (n = 3) and the results presented as mean values.

#### DPPH

The free radical scavenging capacity of samples was determined using the method proposed by Nuutila *et al*. [40]. The results were expressed as Trolox equivalents (TxE). The analysis was repeated (n = 3) and the results presented as mean values.

#### Total polyphenol contents

The total phenolic content was measured in the samples before and after the roasting process using the Folin–Ciocalteu colorimetric method. Absorbance of the resulting blue color was measured at 765 nm with a UV–VIS spectrophotometer. The results were expressed as gallic acid equivalents (GAE). The analysis was repeated (n = 3) and the results presented as mean values.

#### HPLC analysis of phenolic acids and flavonoids

The selected phenolic acids and flavonoids were determined by HPLC methods. The analysis was performed using a Dionex LC system equipped with a photodiode array detector (Dionex). The absorption spectra were recorded in the range of 200–600 nm. The flow rate was 1 mL/min, the column temperature was 30°C, and the injection volume was 20 μL. Qualitative identification was performed by comparing the retention times and spectra with the standards. Simultaneous monitoring was performed at 280 nm.

Separation of selected phenolic acids was performed on a Hypresil Gold (Thermo Electron) C18 column (250 mm × 4.6 mm; 5 μm). The binary mobile phase consisted of 0.1% (v/v) formic acid in water (eluent A) and methanol–acetonitrile (80:20, v/v; eluent B). The gradient program was as follows: 0–5 min (0% B), 7–15 min (10% B), 25 min (25% B), 34 min (65% B), 35–39 min (100% B), 40–45 min (0% B). Chlorogenic acid was determined after extraction without alkaline hydrolysis, and the selected bound phenolic acids after alkaline hydrolysis.

Flavonoids were determined after acidic hydrolysis; separation was performed on a Ascentis (Supelco) C18 column (250 mm × 4.6 mm; 5 μm). The binary mobile phase consisted of 0.1% (v/v) formic acid in water–methanol (75:25, v/v, pH 2.7; eluent A) and 0.1% (v/v) formic acid in methanol (eluent B). The gradient program was as follows: 0–2 min (0% B), 10–20 min (15% B), 30 min (40% B), 35–44 min (100% B), 47–51 min (0% B).

The analysis was replicated (n = 3), and the results given as mean values.

### Sensory analysis

Sensory examination of the aroma for both the coffee substitutes and the coffee was conducted by the profile method using developed glossaries of aroma descriptors (coffee like, roasted, bread like, malty, brown, nutty, chocco, skiny, burnt, acidic, earthy, bitter taste, salty taste, sweet taste, sour taste). Attributes were selected by a panel during preliminary session of brewed samples and according to Stampanoni [41]. Professional panellists of the sensory analysis at Faculty of Food Science and Nutrition (University of Life Sciences, Poznan) participated in this study. All assessors had passed the basic odor test and been trained in sensory analysis at numerous sessions. Their evaluation ability was checked using coffee products. Selected panelists were of age between 25 and 50. The 10 sensory professionals performed aroma profiling of the samples, prepared according to SCAA Standard I Golden Cup, assigning the intensity of each descriptor using a 10-cm graphic scale anchored at 0 to “none” and at 10 to “extremely strong”. Samples were presented to the panel in closed glass vessels. Before sniffing panellist take off the cover from plate. The results from the linear scales were converted into numerical values for data analysis. The results of the analysis were interpreted by applying multidimensional data analysis as part of PCA—principal component analysis [42], using the Analsens 5000E Caret computer system (Systemy Cyfrowe i Oprogramowanie)”

### Statistical analysis

All data were expressed as means ± standard deviations (n = 3). The statistical analyses were conducted using Student’s *t*-test. Values with *p* < 0.05 were considered statistically significant. Statistica 9.0 software (StatSoft, Kraków, Poland) was used for all the analyses.

## Results and discussion

**Fig 1:**
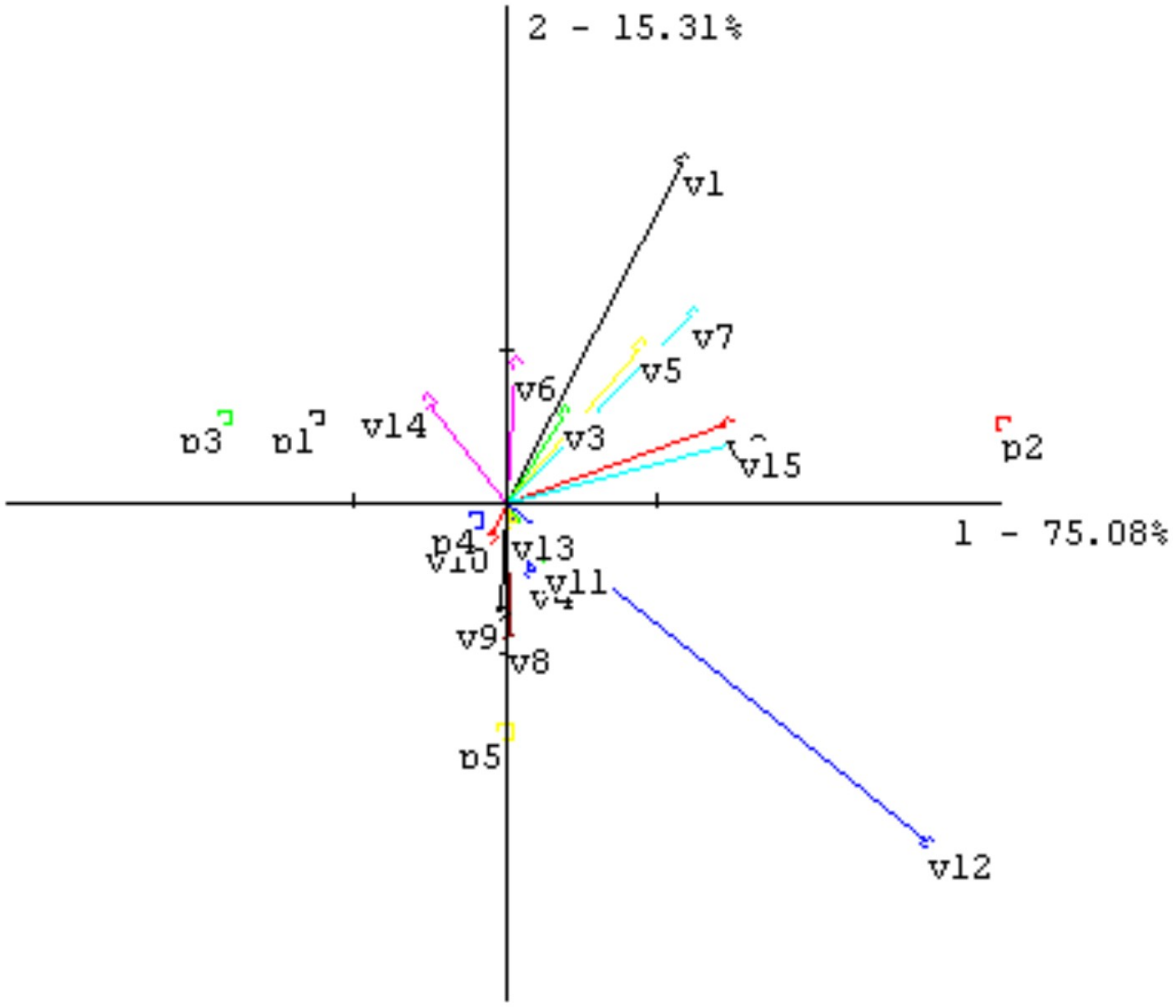
PCA plot of sensory aroma profile data for coffee substitute samples. Sample code; p1—chicory, p2—artichoke, p3—Mix I (10% artichoke), p4—Mix II (20% artichoke) and p5—Mix III (30% artichoke). Descriptors: v1—coffee like, v2—roasted, v3—bread like, v4—malty, v5—brown, v6—nutty, v7—chocco, v8—skiny, v9—burnt, v10—acidic, v11—earthy, v12—bitter taste, v13—salty taste, v14—sweet taste, v15- sour taste.

Neuroactive β-carbolines in food are recently the subject of many studies consideration, so on the basis of our previous research [8], chicory was chosen from among the traditionally used ingredients of coffee substitutes as the one with the highest carboline content; artichoke was chosen from the few newly proposed ingredients using the same criterion. It worth to note chicory is the main component of coffee substitute and sometime used as only one. When roasted, and in the three roasted mixtures (containing 10%, 20%, and 30% artichoke), these materials were characterized through aroma profile analysis of prepared beverages in order to control possibility of applying this material from sensory point of view. A graphical representation of the sensory data is given in Fig 1. The greatest difference was observed between chicory and artichoke, with the artichoke being bitterer in taste. The mixtures with 10% and 20% additions of artichoke were rather similar to chicory: Mix III, with 30% was more distant. On the basis of this experiment, it seems that more than 30% artichoke might not be acceptable.

Both roasted materials and the three mixtures were tested for level of β-carbolines; the results are presented in Table 1. It can be seen that the concentration of harman was significantly higher in artichoke and that its addition to the mixtures resulted in an increased concentration of this compound, and consequently of total β-carbolines—particularly in Mix III. Norharman (Table 1) appeared to be in almost the same concentration in both materials. The estimated concentrations of carbolines in all the materials are relatively high and are comparable with these of true coffee [1]; norharman was found in coffee over very wide range of concentration (1–10 μg/g), so in some cases it might be more common in chicory. In such a case, chicory coffee has to be regarded as a rich source of beta-carbolines in an everyday food, as modern *wellness* product that lacks caffeine, which is not tolerated by everybody. According to Pfau and Skog [43], cereal coffee seems to have the greatest carboline content of the several kinds of food that contain relatively high amounts of these compounds (roasted beef, pork, poultry, fish, and true coffee).

**Table 1:** Concentration of β-carbolines in roasted materials for coffee substitute [μg/g].

β-carboline	Chicory	Artichoke	Mix I	Mix II	Mix III
**Harman**	1.76^a^ ± 0.16	2.90^b^ ± 0.11	1.80^a^ ± 0.04	2.02^c^ ± 0.03	2.30^d^ ± 0.04
**Norharman**	2.90^a^ ± 0.11	3.13^a^ ± 0.18	2.87^a^ ± 0.06	2.86^a^ ± 0.03	2.83^a^ ± 0.10

LOD: 0.06 μg/g harman, 0.1 μg/g norharman,; LOQ: 0.12 μg/g harman, 0.20 μg/g norharman

^a, b, c, d^—results with different lowercase letters are significantly different (P < 0.05)

Mix I, Mix II, Mix III–mixture of chicory/artichoke with 10, 20 and 30% of artichoke relatively

Nevertheless, chicory coffee—like all roasted materials—also contains other bioactive compounds resulting from heat treatment, and these are usually not healthful. Their concentration levels and the proportion of healthful and harmful constituents need to be better investigated. It is an interesting question whether the addition of artichoke increases or decreases the level of undesirable compounds.

One of these components is furan, often accompanied by methyl furan; the concentrations of these were tested in all samples. Altogether, the five identified furans are presented in Table 2.

**Table 2:** Concentration of furan and it’s derivatives (furanic compounds) in roasted materials for chicory coffee [μg/g].

Furanic compound	Chicory	Artichoke	Mix I	Mix II	Mix III
**Furan**	0.39^a^ ± 0.05	0.62^b^ ± 0.05	0.34^a^ ± 0.02	0.35^a^ ± 0.02	0.26^c^ ± 0.02
**2-methylfuran**	2.33^a^ ± 0.26	3.35^b^ ± 0.26	1.72^c^ ± 0.07	1.81^c^^,^^d^ ± 0.08	2.00^a^^,^^d^ ± 0.10
**2-penthylfuran**	1.08^a^ ± 0.10	1.05^a^^,^^c^ ± 0.17	0.9^b^^,^^c^ ± 0.03	1.26^d^ ± 0.04	0.77^c^ ± 0.03
**2,5-dimethylfuran**	1.73^a^ ± 0.05	2.04^b^ ± 0.08	1.55^c^ ± 0.08	1.86^a^ ± 0.12	1.77^a^ ± 0.04
**2,2 bifuran**	9.24^a^ ± 0.05	4.69^b^ ± 0.57	4.27^c^± 0.09	4.99^b^ ± 0.10	2.33^d^ ± 0.14
**2-furanyl-5-methylfuran**	2.02^a^ ± 0.07	1.00^b^^,^^c^ ± 0.15	0.82^b^^,^^c^ ± 0.09	0.95^c^ ± 0.02	0.47^d^ ± 0.02
**Furfural**	128.19^a^ ± 0.89	38.87^b^ ± 0.78	103.01^c^ ± 0.20	99.24^c^ ± 0.51	43.05^b^ ± 0.64

LOD for furan 0.07 μg/g (0.003 μg/mL), LOQ for furan 0.21 μg/g (0.008 μg/mL)

^a, b, c, d^- results with different lowercase letters are significantly different (P < 0.05)

Mix I, Mix II, Mix III–mixture of chicory/artichoke with 10, 20 and 30% of artichoke relatively

It is evident that furan and methyl furan occurred in much higher concentrations in artichoke than in chicory. However, it is much more interesting that the mixtures, where we expected to observe higher amounts of furans than in in chicory, actually contained even lower concentrations. The only possible explanation for this is that they were lost as volatiles by evaporation during the process of mixing the ground materials. This loss would be difficult to avoid, but in any case leads to a positive outcome.

The levels of furan (0.3 μg/g or 15 ng/mL in chicory; 0.6 μg/g or 24 ng/mL in artichoke, if assumed for approximate calculation, that 100% would be extracted) are not as high as those reported in the literature for coffee—e.g., 0.885 μg/g [19] or 51.7 ng/g of brewed coffee [11]. The furan levels in brewed coffee have not be directly compared because the samples of coffee were not prepared from the same recipe or using the same kind of coffee (which differed by variety and roasting intensity). The comparison might thus be only approximate. However, the other furans (Table 2) detected in both materials occurred in high amounts—higher than those reported by Fromberg *et al*. [19]. In particular, a very high concentration was found for bifuran (highest in chicory at 9.24 μg/g), which was not mentioned by that author for coffee, but which has been identified in coffee of various origins by Yener *et al*. [27]. The concentration of total furans in chicory and artichoke was about 40 times the concentration of furan itself. Despite the high concentration of furan in chicory and artichoke, it seems that neither contains as much furan as true coffee. Table 2 also shows furanic compounds such as furfural—this occurs in large amounts, mostly in chicory, while HMF was not detected. This is not unusual: Yener *et al*. [27] have also found furfural, but not HMF, in coffee.

As seen in Table 3, the level of CML in artichoke is higher than in chicory, and the addition of artichoke caused a significant increase in CML in the mixtures; however, the difference is not very high. Generally, the level of CML concentration is highest of all the investigated compounds, at about ten times higher than total furans and total carbolines. The level of CML found here is far above that described by Loaëc *et al*. [30], but CML occurs in various heat-treated foods that are consumed in the daily diet—such as bread, which can contain about 49 μg/g in the crust and 15 μg/g in the crumb [15], sometimes reaching as much as 80 μg/g in the crust and 35 μg/g in the crumb [44].

**Table 3:** Concentration of carboxymethyllysine and acrylamide in roasted materials for chicory coffee [μg/g].

Compound	Chicory	Artichoke	Mix I	Mix II	Mix III
**Carboxymethyllysine (CML)**	85.29^a^ ± 0.18	114.14^b^ ± 0.29	102.80^c^ ± 0.08	107.32^c^ ± 0.08	109.69^c^ ± 0.27
**Acrylamide (ACR)**	0.523^a^ ± 0.05	17.08^b^±1.71	1.85^c^±0.18	2.51^d^±0.25	6.13^e^±0.61

CML: LOD 0.42 ng/kg, LOQ 1.29 ng/kg; ACR: LOD 0.9 μg/kg, LOQ 3 μg/kg

^a, b, c, d, e^- results with different lowercase letters are significantly different (P < 0.05)

Mix I, Mix II, Mix III–mixture of chicory/artichoke with 10, 20 and 30% of artichoke relatively

According to data obtained, the amount of CML in a 250-mL glass of cereal coffee, prepared according to the label directions, is about 1200 μg; the literature suggests that 100 g of bread contains no less than 3000 μg. Nevertheless, chicory must be regard as a source of CML and the addition of artichoke increased the level of this compound in the mixtures. Table 3 also presents data on acrylamide. Like CML, its concentration in artichoke dramatically exceeds that in chicory, thus exerting a significant effect on the ACR contents of the mixtures. In the 2018 European Commission recommendations [45], the indicative value for acrylamide in coffee substitutes made from chicory is 4000 μg/kg: in our study, chicory alone and Mixes I and II fall below this threshold but Mix III highly overcome it.

The mechanism of antioxidant activity of the MR products is still unclear, so a complex system such as food requires more than one method to evaluate AOA [34]. The same holds for the roasted raw materials used in chicory coffee. The antioxidant activity of the products in terms of TS, ABTS, and DPPH (Table 4) was lower in artichoke than in chicory, but the addition of artichoke to the studied mixtures did not significantly changed the AOA in relation to chicory: this was most evident in Mix III. The AOA values of all the measured products are relatively high—e.g., ABTS is comparable to the value reported by Komes *et al*. [46] for roasted chicory and almost two times higher than in cookies cooked under the hottest and longest regime [36].

**Table 4:** Antioxidant properties (AOA) of raw material for chicory coffee.

Antioxidant property	Chicory	Artichoke	Mix I	Mix II	Mix III
**Antioxidant activity**					
**TP [mg GAE /g]**	34.26^a^ ± 2.19	28.64^b^ ± 1.88	33.83^a^ ± 2.17	33.10^a^ ± 2.13	32.35^a^ ± 2.08
**ABTS**^**+**^ **[mg TxE / g]**	61.51^a^ ± 0.33	55.06^b^ ± 0.67	60.90^a^ ± 0.40	60.13^a^ ± 0.08	59.30^a^ ± 0.03
**DPPH [mg TxE /g]**	35.30^a^ ± 2.26	29.70^b^ ± 1.90	34.78^a^ ± 2.22	34.06^a^ ± 2.18	33.52^a^ ± 2.14
**Free phenolic acids [μg/g]**					
**chlorogenic**	171.7^a^ ± 0.4	111.2^b^ ± 7.1	160.4^c^ ± 5.3	151.9^c^^,^^d^ ± 8.5	148.4^d^ ± 7.2
**Bound phenolic acids [μg/g]**					
**caffeic**	700.5^a^ ± 47.6	837.0^b^ ± 56.0	716.6^a^ ± 5.4	724.9^a^ ± 7.4	745.5^c^ ± 5.4
**coumaric**	11.5^a^ ± 0.3	23.4^b^ ± 1.2	12.8^a^ ± 0.1	13.8^a^^,^^c^ ± 0.4	15.2^c^ ± 0.3
**ferulic**	30.2^a^ ± 0.3	6.6^b^ ± 0.4	28.0^a^ ± 0.6	25.2^a^^,^^c^ ± 1.5	22.8^c^± 1.0
**syringic**	nd	9.1^a^ ± 0.3	0.9^b^ ± 0.01	2.0^c^ ± 0.2	2.8^d^ ± 0.1
**Flavonols [μg/g]**					
**quercetin**	7.72^a^ ± 0.14	0.95^b^ ± 0.13	7.07 ^a^± 0.54	6.19^c^ ± 0.52	5.70^c^ ± 0.26
**Flavones [μg/g]**					
**luteolin**	9.85^a^ ± 1.75	nd	9.13^a^ ± 0.42	8.28^b^ ± 0.66	7.17^c^ ± 0.67
**apigenin**	9.06^a^ ± 0.02	4.46^b^ ± 0.34	8.00^c^ ± 0.93	8.10^c^ ± 2.13	7.85^c^ ± 1.19

^a, b, c, d^- results with different lowercase letters are significantly different (P < 0.05), nd—not detected LOD 0.1 μg/g

Mix I, Mix II, Mix III–mixture of chicory/artichoke with 10, 20 and 30% of artichoke relatively, TxE-trolox equivalent, GAE- gallic acid equivalent

This might be regarded as a positive aspect of cereal coffee quality. Especially, that several phenolic compounds have been demonstrated to possess strong inhibitory effects on Maillard reaction products [15]. Table 4 also shows a higher concentration in chicory of antioxidant compounds such as chlorogenic acid, quercetin, and the flavones luteolin and apigenin. Only some bound phenolic acids were present on a slightly higher level in artichoke.

On the basis of data obtained in this study the total β-carbolines per glass of beverage (250 mL) prepared from Mix III may amounted to 64.13 μg, while CML 1371 μg, ACR 76.62 μg and furan 3.25 μg. Antioxidant activity of such portion of brew expressed by TP, ABTS and DPPH reached 404.37 mg GAE, 741.25 mg TxE and 419 mg TxE respectively.

Taking into account all the measured bioactive components in the two selected coffee substitute materials, and adopting the positive and negative indices of Cheng *et al*. [35], it can be stated that furans, CML, and ACR are undesirable, while AOA is desirable. The question arises about β-carbolines. In the case when β-carbolines neuroactivity would be documented as positively acted for human health, chicory and artichoke might be proposed as rich source of them. However, it is important to keep in mind that coffee substitutes contain also other components like CML, ACR and furans, which demonstrate adverse human health effects. But in the case of possible neurotoxic effect of β-carbolines, coffee substitutes better should not be used in much amount. This still needs to be clarified.

## Conclusions

Chicory and artichoke contained high levels of β-carbolines. Artichoke, a new proposed ingredient in coffee substitutes, appears to be a richer source of β-carbolines than the traditionally chicory, and its addition increases the concentration of β-carbolines. Both materials contained high level of undesirable components, such as furan and its derivatives, CML and particularly acrylamide, much higher in artichoke. This observation might be extended to all cases when looking for any new bioactive raw materials. Together with desirable substance some harmful components may be introduced even on unexpected high level.
